# Mutational Analysis of the Cyanobacterial Nitrogen Regulator PipX

**DOI:** 10.1371/journal.pone.0035845

**Published:** 2012-04-30

**Authors:** Karim Boumediene Laichoubi, Javier Espinosa, Miguel Angel Castells, Asunción Contreras

**Affiliations:** División de Genética, Universidad de Alicante, Alicante, Spain; Belgian Nuclear Research Centre SCK/CEN, Belgium

## Abstract

PipX provides a functional link between the cyanobacterial global transcriptional regulator NtcA and the signal transduction protein P_II_, a protein found in all three domains of life as integrators of signals of the nitrogen and carbon balance. PipX, which is toxic in the absence of P_II_, can form alternative complexes with NtcA and P_II_ and these interactions are respectively stimulated and inhibited by 2-oxoglutarate, providing a mechanism by which P_II_ can modulate expression at the NtcA regulon. Structural information on PipX-NtcA complexes suggests that PipX coactivates NtcA controlled genes by stabilizing the active conformation of NtcA bound to 2-oxoglutarate and by possibly helping recruit RNA polymerase. To get insights into PipX functions, we perform here a mutational analysis of pipX informed by the structures of PipX-P_II_ and PipX-NtcA complexes and evaluate the impact of point mutations on toxicity and gene expression. Two amino acid substitutions (Y32A and E4A) were of particular interest, since they increased PipX toxicity and activated NtcA dependent genes in vivo at lower 2-oxoglutarate levels than wild type PipX. While both mutations impaired complex formation with P_II_, only Y32A had a negative impact on PipX-NtcA interactions.

## Introduction

Cyanobacteria are phototrophic organisms that perform oxygenic photosynthesis. Autotrophic growth requires the constant assimilation of ammonium via the GS-GOGAT cycle, resulting in consumption of 2-oxoglutarate [1], [2] that accumulates during nitrogen starvation, making this metabolite an excellent indicator of the intracellular carbon to nitrogen balance [3], [4]. 2-oxoglutarate, the signal of nitrogen deficiency, modulates the activity and/or binding properties of three key cyanobacterial nitrogen regulators: the signal transduction protein P_II_, the transcriptional activator NtcA, and the regulatory factor PipX. The homotrimeric P_II_ protein is one of the most conserved and widespread signal transduction protein in nature and plays key roles in nitrogen assimilatory processes [5]. P_II_ proteins, which contain three binding sites (one per subunit) for 2-oxoglutarate and ATP, regulate by direct protein-protein interactions the activity of proteins implicated in nitrogen metabolism (reviewed in [6], [7]). The first two P_II_ receptors identified in cyanobacteria were the enzyme N-acetyl-L-glutamate kinase (NAGK), a P_II_ target conserved across domains of life during the evolution of oxygenic photosynthetic organisms [8], [9], [10], and the regulatory factor PipX [8], [11]. Structural and functional details are known for the P_II_-NAGK and P_II_-PipX complexes [12], [13]. The P_II_-NAGK complex consists of two polar P_II_ trimers sandwiching one ring-like hexameric NAGK, with the flexible T-loop, a key element for regulatory interactions_,_ adopting a novel compact shape. In the PipX-P_II_ complex, one P_II_ trimer with its T-loops in a vertically extended conformation sequesters three PipX molecules. These consists of a tudor-like domain, mediating all contacts with P_II_, and two C-terminal helices [13], [14]. ATP in concert with 2-oxoglutarate prevents complex formation of P_II_ with either NAGK or PipX [11], [15] while ADP stimulates P_II_-PipX complex formation [13], [16].

In cyanobacteria, multiple metabolic and developmental processes are induced by nitrogen starvation. NtcA, the global regulator for nitrogen control, activates genes involved in nitrogen assimilation, heterocyst differentiation and acclimation to nitrogen starvation [17], [18], [19]. 2-oxoglutarate stimulates binding of NtcA to target sites [20], transcription activation *in vitro* [21] and complex formation between NtcA and PipX [11]. The interaction between PipX and NtcA is known to be relevant under nitrogen limitation for activation of NtcA-dependent genes in *S. elongatus* and *Anabaena* [11], [22], [23]. The PipX-NtcA complex consist of one active (2-OG bound) NtcA dimer, and two PipX molecules. Each NtcA subunit binds one PipX molecule in such a way that it stabilizes the active NtcA conformation and probably helps recruiting RNA polymerase without providing extra DNA contacts [13], [24].

The 2-oxoglutarate dependent partner swapping of PipX between P_II_ and NtcA [11] provides a mechanistic link between P_II_ signaling and gene expression. When nitrogen is abundant the intracellular levels of 2-oxoglutarate are low and sequestration of PipX by P_II_ decrease NtcA-PipX complex formation. The tudor-like domain of PipX provides the contacts regions within NtcA-PipX and P_II_-PipX complexes. The structure of P_II_-PipX complex suggests regulatory potentialities [13].

While P_II_ is essential in *S. elongatus*, point mutations at *pipX* or genetic manipulations resulting in a lower level of PipX protein suffice to overcome the lethality of *glnB* mutants [25], [26], [27], supporting the view that PipX is toxic and that this toxicity is counteracted by P_II_. It is not known whether PipX toxicity results from an excess of NtcA activity or from the interaction of PipX with additional targets or processes. So far, we have been unable to obtain genetic evidence supporting the implication of NtcA in toxicity [25].

To deepen our understanding of PipX functions, we analyzed here a collection of site-directed point mutations at PipX for their effects on partner binding, toxicity, and gene expression. As a result, we discovered that E4A and Y32A, which in yeast two-hybrid assays impair complex formation with P_II_, or with both P_II_ and NtcA, respectively, were gain-of-function mutations that increased PipX toxicity and gene expression at low 2-oxoglutarate levels.

## Results

### Effect of Substitutions at PipX on Interactions with P_II_ and NtcA: Rational for Site-directed Mutagenesis

**Figure 1:**
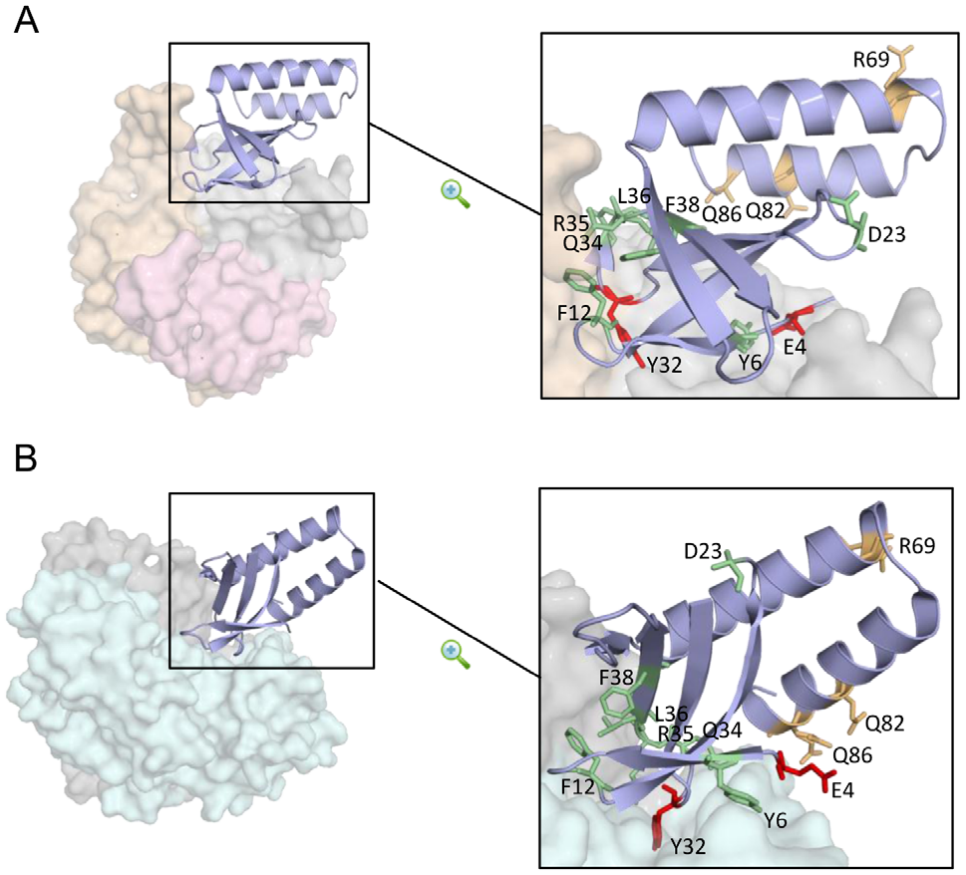
Localization of PipX residues discussed in this work. Details of interactions between one PipX monomer and P_II_ (**A**) **or NtcA** (**B**) **are shown in the P_II_-PipX (PDB file 2XG8) and NtcA-PipX complexes (PDB file 2XKO).** PipX structures are represented in blue ribbon while surface representation and pale colours are used for P_II_ (subunits in grey, yellow and pink) and NtcA (grey and cyan). In each case, the enlargement of PipX to the right shows relevant side chains of residues mutated to alanine (except Q34, mutated to glutamate). Mutated residues that increase, decrease or do not affect PipX toxicity are colored in red, green, and gold, respectively.

To get insights into the functions of *S. elongatus* PipX, a mutational analysis was performed. The idea was to evaluate the *in vivo* impact of mutations on known PipX functions, that is, PipX toxicity and coactivation of NtcA dependent genes. The same type of analysis was performed in a previous work [26] for the two known spontaneous point mutations at the *pipX* coding region (substitutions R54C and L65Q, targeting the C-terminal helices of PipX). Since structural knowledge of PipX complexes is now available, here we focus on PipX surfaces that are relevant for the known complexes with NtcA and PipX. Site-directed mutagenesis was informed by the structures of the PipX-P_II_ and PipX-NtcA complexes [13]. Mutations targeted residues of the PipX surface involved in contacts with P_II_ and NtcA (Y6, F12, Y32, Q34, R35, L36), contacts with just P_II_ (E4), just NtcA (Q86) or residues chosen on the basis of position and/or sequence conservation (D23, F38, R69, Q82). The positions of targeted residues on the structures of PipX as found in complexes with P_II_ and NtcA are shown in Fig. 1. A stereo view of PipX in each of the two complexes is provided in Fig. S1.

**Figure 2:**
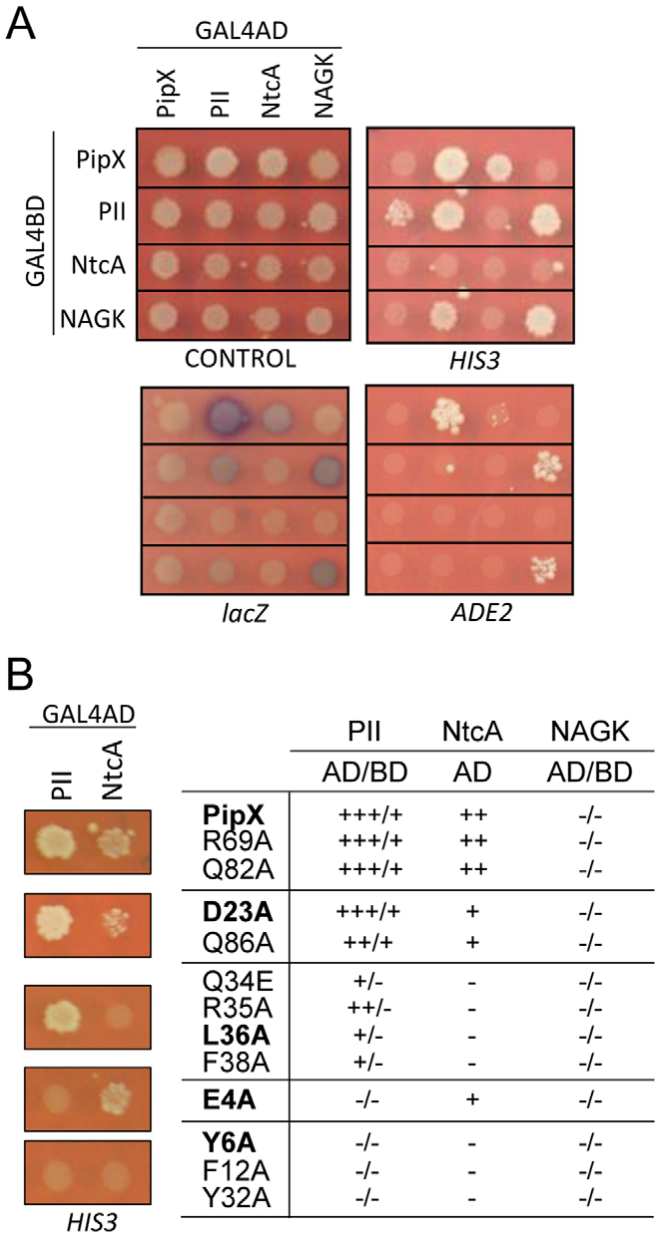
Effect of PipX substitutions in yeast two hybrid interaction signals. (A) An illustrative yeast two hybrid Interaction assay involving wild type PipX, P_II_, NtcA and NAGK proteins fused to either GAL4AD and GAL4BD. Photographs show growths on diploid media (CONTROL), on histidine (*HIS3*) and adenine (*ADE2*) minus media, and x-gal test (*lacZ*) on a diploid plate. (B) Yeast two hybrid interaction pattern of PipX point mutants. Growth of diploids carrying GAL4AD:P_II_ or GAL4AD:NtcA and the wild type (PipX) or the BD:PipX substitutions indicated in bold type (representative for each group of proteins), on minus histidine medium. The table summarizes the level of interaction signals between fusions of GAL4AD and GAL4BD to PipX derivatives and their complementary domains fused to P_II_, NtcA and NAGK (negative control), as indicated. Signs (from +++ to -) indicate levels of yeast two-hybrid expression from *HIS3*, *ADE2* and *lacZ* reporters according to previously described conventions [8].

The yeast two-hybrid system was used to analyze the effect of the different amino acid substitutions at PipX on protein-protein interactions with NtcA and P_II_. In previous works we showed the specificity of interactions mediated by PipX [11] and discussed the advantages, as well and limitations, of the yeast two-hybrid system in the study of interactions mediated by nitrogen regulators [8], [28], [29], [30], [31]. Pairs of plasmids encoding fusions of GAL4AD and GAL4BD to each of the PipX mutant derivatives were generated and tested for signals with the corresponding P_II_ and NtcA yeast two-hybrid fusions (Fig. 2A). Expression of *HIS3, ADE2* and *lacZ* reporters, determined in Y187/PJ696 diploids as previously described [8], allowed grouping of the mutations into different interactions patterns (Fig. 2B). For the sake of simplicity, results have been grouped into 5 different classes. Only wild type controls and data corresponding to one representative example of each type of interaction pattern and of one reporter (*HIS3*) are shown. Additional yeast two-hybrid data are provided in Fig. S2.

Control substitutions R69A and Q82A had no effect on interactions signals, while the remaining mutations perturbed yeast two-hybrid interactions to varying extents. Mutations D23A and Q86A had no detectable impact on signals with P_II_, and very little effect on signals with NtcA. Mutation E4A specifically impaired signals with P_II_, being the only one with this interaction pattern. The remaining mutations, all targeting residues located in the overlapping surface of PipX-P_II_ and PipX-NtcA complexes affected interactions with both P_II_ and NtcA, although two different interaction patterns could be distinguished: Y6A, F12A and Y32A completely abolished all interaction signals, while Q34E, R35A, L36A and F38A still gave signals with P_II_ (when pairing GAL4AD-P_II_ and GAL4BD-PipX derivatives).

### Effect of Point Mutations on PipX Toxicity in the Presence of P_II_

**Figure 3:**
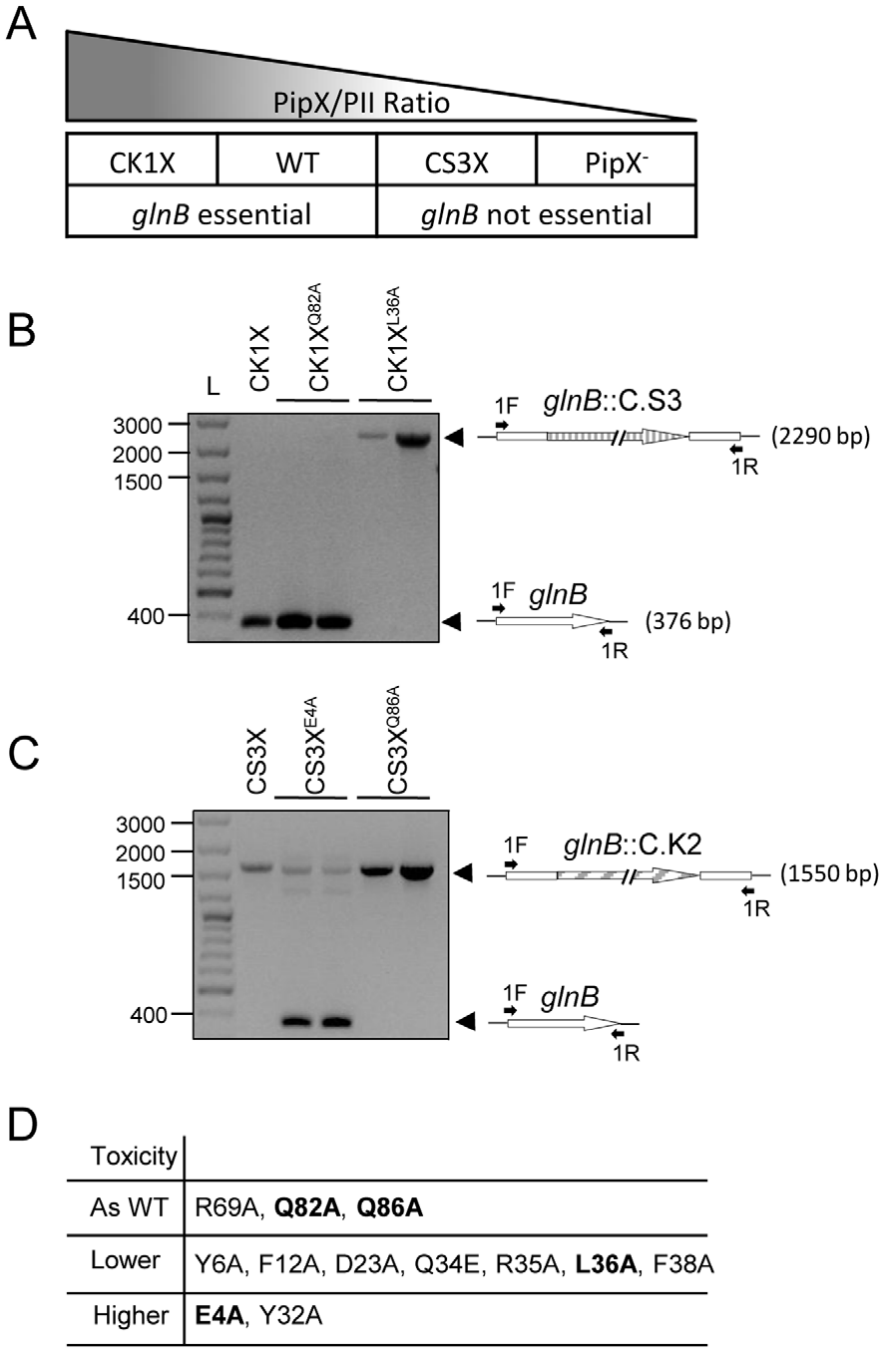
Effect of *pipX *mutations on toxicity. (A) Cartoon illustrating the correspondence between different genetic backgrounds for *pipX* and the PipX/P_II_ ratio (triangle) decreasing from left to right. PCR analyses for detection of *glnB* alleles in CK1X (B) and CS3X (C) strains and derivatives. Transformant clones of the indicated strains carrying compatible C.K1 or C.S3 insertions in *glnB* were analyzed by PCR after three consecutive transfers onto selective media. Two independent clones for each selected point mutation derivative are shown. Positions of PCR products are indicated to the right. The corresponding wild type (*glnB*) and mutant alleles (*glnB*::C.S3 and *glnB*::C.K2) are schematically represented with indication of the positions of the PCR primers used (black arrows). Lane L, size marker (GeneRuler 100 bp plus DNA ladder, Fermentas). (D) Classification of PipX substitutions into three toxicity levels based in the PCR analysis. Substitutions indicated in bold type correspond to the representative examples illustrated in B and C.

Previously, we established that replacing *pipX* by the marker fusions Φ(C.S3-*pipX*) and Φ(C.K1-*pipX*) resulted in strains with a reproducible decrease (CS3X) and increase (CK1X), respectively, on the levels of the PipX protein, but no significant differences in viability with respect to wild type *S. elongatus* cultures ([26] and data not shown). We found that in both wild type and CK1X strains *glnB* could not be completely inactivated, whereas in CS3X and in strains in which *pipX* was previously inactivated, or carried partial loss-of-function mutations, *glnB* could be easily inactivated [25], [26], [27], a result confirmed with wild type *S. elongatus* cultures from different sources. A simple interpretation of these observations (Fig. 3A) is that a high PipX/P_II_ ratio results in lethality and thus inactivation of *glnB* is viable only when PipX levels (or activity levels) are lower than in the wild type.

Since increasing the PipX/P_II_ ratio above wild type levels increases lethality, we wondered on the effect of increasing the expression of PipX the different PipX mutant proteins above the wild type levels, and thus attempted construction of *S. elongatus pipX* mutant derivatives in the CK1X background. Kanamycin-resistant strains carrying completely segregated Φ(C.K1-pipX*) alleles (the asterisk symbolizes each one of the mutations tested) were obtained in 10 out of the 12 cases studied. However, no viable kanamycin-resistant transformants carrying the mutant alleles Φ(C.K1-*pipX^E4A^*) or Φ(C.K1-*pipX^Y32A^*) could be recovered after repeated transformation attempts, strongly suggesting that the corresponding mutations increased PipX toxicity in *S. elongatus*.

### Effect of Point Mutations on PipX Toxicity in the Absence of P_II_

To identify *pipX* mutations suppressing PipX toxicity in the absence of P_II_, the inactive allele *glnB*::C.S3 was introduced by homologous recombination into the 10 strains already carrying Φ(C.K1-pipX*) alleles. Subsequent PCR analysis (Fig. S3) of the streptomycin-resistant transformant clones carrying the different Φ(C.K1-pipX*) derivatives allowed distinction of two groups: one that behaved as the CK1K control (CK1X^R69A^, CK1X^Q82A^ and CK1X^Q86A^), that is, wild type *glnB* alleles were easily amplified in these strains, and another (expressing mutations Y6A, F12A, D23A, Q34E, R35A, L36A and F38A) that did not. Since short PCR fragments are always more efficiently amplified with the same pair of primers, the failure to detect the shorter *glnB* alleles in this analysis is a good indication of its absence from cultures (complete inactivation). In addition, the inactive allele *glnB*::C.S3 was detected in all clones were amplification of the shorter *glnB* alleles failed. Fig 3B shows PCR results obtained with the control and one representative example of each of the two groups (lanes CK1X, CK1X^Q82A^ and CK1X^L36A^). Schematic representations of the wild type (*glnB*) and inactive alleles (*glnB*::C.S3) detected in the PCR are included for the sake of clarity. According to this analysis, the PipX mutations were categorized as being as toxic as or more toxic than wild type PipX (Fig. 3D). This information is also incorporated on Fig. 1.

A genetic test was carried out to determine whether mutations increasing PipX toxicity while impairing binding to P_II_ could still be counteracted by P_II_. To express the *pipX* mutations we now used the Φ(C.S3-*pipX*) construct. The idea was to verify previous results obtained with the Φ(C.K1-*pipX*) construct and to look for mutations increasing toxicity that may have escaped previous detection. With this in mind, strains expressing PipX derivatives in the form Φ(C.S3-*pipX*) were generated for those site-directed mutations found to be as toxic (R69A, Q82A and Q86A) or more toxic (E4A and Y32A) than PipX in our previous analyses. Subsequently, the viability of inactivating *glnB* with the C.K2 compatible marker was investigated. PCR analysis of the kanamycin-resistant transformant clones generated in control (CS3X) and mutant strains were performed in all cases. Representative examples (lanes CS3X, CS3X^E4A^ and CS3X^Q86A^) are shown in Fig 3C.

Complete segregation of the *glnB*::C.K2 allele took place in strains CS3X, CS3X^R69A^, CS3X^Q82A^ and CS3X^Q86A^, indicating that the corresponding mutations do not appreciably increase PipX toxicity. However, strains CS3X^E4A^ and CS3X^Y32A^ did retain wild type *glnB* alleles after several consecutive transfers onto selective plates, a result indicating that the toxicity of PipX^E4A^ and PipX^Y32A^ was counteracted by the presence of P_II_ and providing independent support for the higher toxicity conferred by mutations E4A and Y32A (Fig. 3D).

### PipX Toxicity and Expression of NtcA Dependent Genes

**Figure 4:**
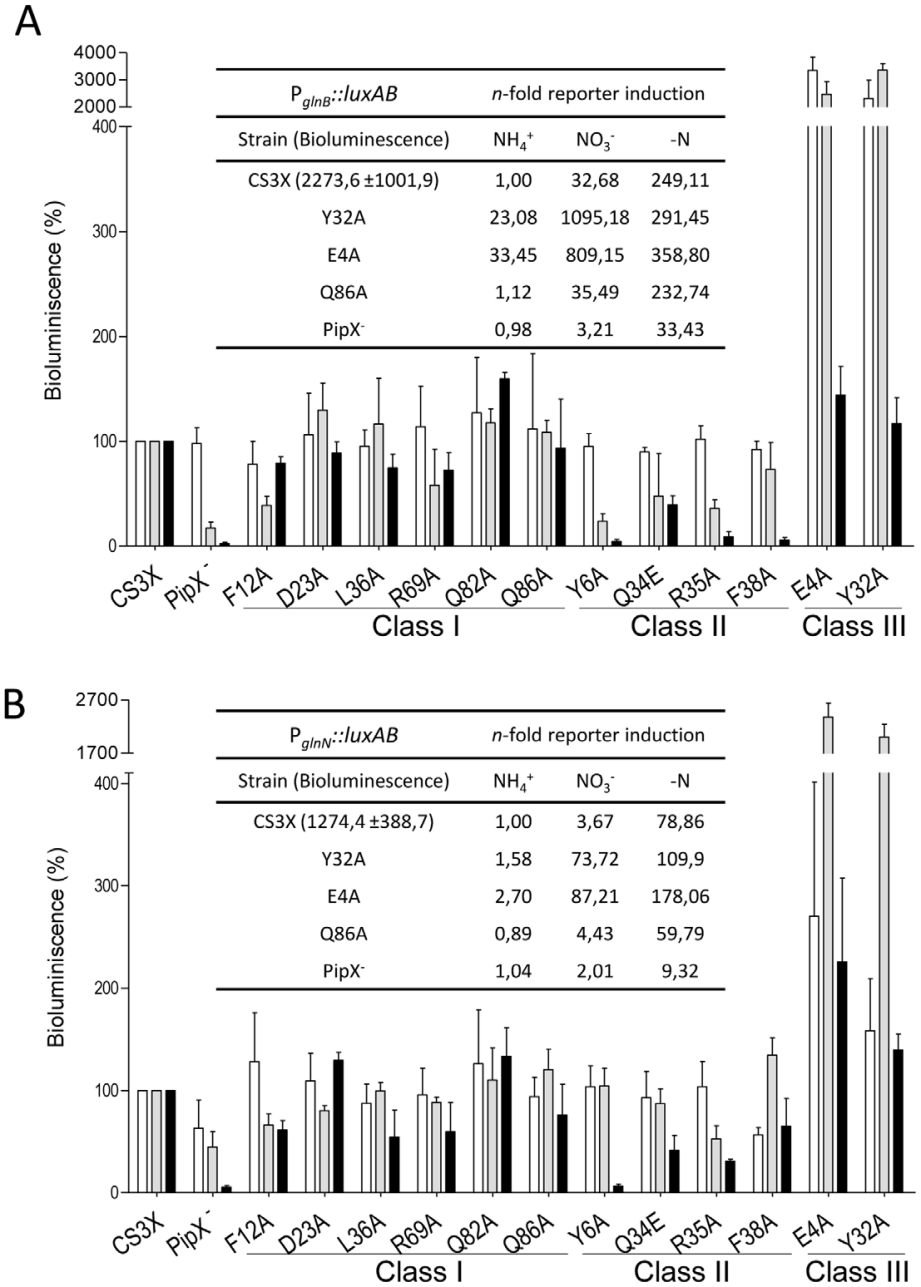
Effect of *pipX *mutations on P*glnB*::*luxAB *(A) and P*glnN*::*luxAB *(B) expression. Bioluminescence relative to the levels in the control strain (CS3X) were determined in cells grown in the presence of ammonium (white bars), nitrate (grey bars) and 24 h after cultures were shifted from ammonium-containing to nitrogen-depleted medium (black bars). Means ± SD values from at least 3 independent experiments are plotted. *pipX* mutant (PipX^-^), control strain (CS3X) and point mutants (derived from CS3X) are indicated. The inset shows the absolute bioluminescence values for CS3X grown in ammonium and the induction of these values under different nitrogen regimens and/or selected genetic backgrounds.

Because the previously investigated spontaneous point mutations that suppressed PipX toxicity in P_II_ deficient strains did have a negative impact on NtcA coactivation [25], [26], we wondered whether these two features could be genetically separated. With this in mind, the impact of point mutations on PipX function as NtcA co-activator was analysed as before [26], that is, in CS3X* strains containing the NtcA-dependent promoter derivatives P*_glnB_::luxAB* and P*_glnN_::luxAB*. To determine reporter expression, bioluminescence was measured from cultures grown to mid-exponential phase in the presence of ammonium or nitrate and after cultures were shifted from ammonium-containing to nitrogen-depleted medium. Results are summarized in Fig. 4. For simplicity, results are represented relative to the control in the same conditions (bars) and mutants were grouped into three major classes according to their activity levels. Basal activity levels from the wild type control grown in ammonium are given. Induction ratios (relative to these wild type ammonium cultures) are only shown for selected strains.

A first group of mutants (Class I) was not apparently affected in reporter activation. In addition to mutations that did not affect PipX toxicity (R69A, Q82A and Q86A), this group also included three mutations reducing toxicity (D23A, F12A and L36A), thus providing the first suggestions of genetic separation between toxicity and the co-activation function of PipX.

The four class II mutations, impairing reporter activation, also suppressed toxicity. All mutations affected each of the two reporters, but not necessarily to the same extent, and this was seen under nitrate (grey bars) and nitrogen-free regimes (black bars). The decrease on luciferase values were generally higher with P*_glnB_::luxAB* than with P*_glnN_::luxAB*. Mutation Y6A had the highest impact on luciferase values from each of the two reporters and this effect was almost the same as that observed with the *pipX* null strain used as control (compare black bars, that is conditions of maximal activation, between Y6A and PipX^-^). Mutations R35A and F38A also failed to activate P*_glnB_::luxAB* under nitrogen starvation and significantly reduced activation from P*_glnN_::luxAB*. Mutation Q34E did not produced a drastic effect on activation, but significantly affected activity from both reporters.

Class III mutants (CS3X^E4A^ and CS3X^Y32A^) showed increased levels of reporter activation for both P*_glnB_::luxAB* and P*_glnN_::luxAB* in all three conditions, but the most dramatic effects were on nitrogen containing media. When activity levels of CS3X^E4A^ or CS3X^Y32A^ were compared to CS3X, more than 20 fold differences were obtained with the P*_glnB_::luxAB* reporter for both ammonium and nitrate conditions (white and grey bars, see also induction ratios within the inset tables), and with P*_glnN_::luxAB* for the nitrate cultures (grey bars). For both mutants, activity levels with the later reporter were also higher than for CS3X in ammonium (2.7 and 1.5 times for CS3X^E4A^ and CS3X^Y32A^, respectively), although the differences were not so dramatic. While in the CS3X control very high induction of both reporters was only observed after nitrogen starvation of cultures, in the mutant strains it also happened with nitrate cultures. Furthermore, in the case of the P*_glnB_::luxAB* reporter, the activity levels of mutant strains cultured with nitrate were several times higher than the obtained with any of the nitrogen-starved wild type cultures.

**Figure 5:**
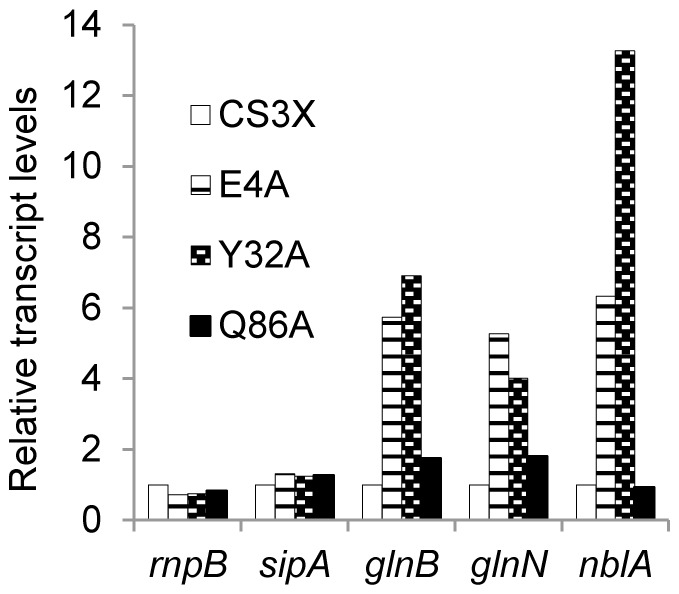
Real-time RT-PCR for *rnpB*, *sipA*, *glnB*, *glnN* and *nblA* genes. Cultures of CS3X and derivatives (for which relevant mutations are indicated) were grown in the presence of nitrate. Transcription levels were calculated relative to the control strain amount (set at 1). Representative data from two independent experiments carried out by duplicate are shown.

The reporter analysis indicated that gene expression from NtcA dependent promoters P*_glnN_::luxAB* and P*_glnB_::luxAB* was up-regulated in the presence of added nitrogen in both CS3X^E4A^ and CS3X^Y32A^ strains. To strengthen this finding we extracted RNAs from nitrate cultures of mutant and control strains and performed quantitative real time PCR (qPCR) analysis for the same (*glnB* and *glnN*) and additional transcriptional units, including *nblA*, a gene known to be activated by NtcA and PipX [22], and two genes which are not regulated by NtcA (*sipA*, and *rnpB*, considered constitutive). The results, shown in Fig. 5, indicated that transcripts from all three NtcA-dependent genes, but not transcripts from the two NtcA-independent genes, are present at higher levels in CS3X^E4A^ and CS3X^Y32A^ than in the two control strains (CS3X and CS3X^Q86A^). The differences ranged between 4 to 7 times for *glnB* and *glnN* transcripts, thus confirming the inferences from the analysis with P*_glnN_::luxAB* and P*_glnB_::luxAB* reporters. The increase in transcript levels for the *nblA* gene was even higher, 6-fold and 13-fold for CS3X^E4A^ and CS3X^Y32A^, respectively.

**Figure 6:**
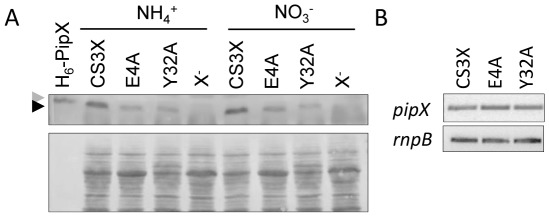
Effect of point mutations on PipX levels. (A) Immunodetection of PipX from strains expressing wild type PipX (CS3X) and the indicated PipX point mutation derivatives (Y32A, E4A) alongside a *pipX* null strain (X^-^) using an Anti-PipX antibody. Detection of endogenous PipX and recombinant H_6_-PipX (10 ng loaded in a control lane) is indicated with black and grey arrowhead, respectively. 60 µg of protein extract were loaded per lane. A protein loading and transfer quality control is shown on the bottom panel. (B) Amplification of *pipX* and *rnpB* (used as a loading control) by RT-PCR in the CS3X and point mutant derivatives (E4A and Y32A) grown in the presence of nitrate. A representative experiment from two independent RNA extractions is shown.

To exclude the possibility that the gain of function phenotypes of CS3X^E4A^ and CS3X^Y32A^ were the result of higher accumulation of these PipX mutant proteins, we investigated *pipX* gene expression in CS3X, CS3X^E4A^ and CS3X^Y32A^ cultures. As shown in Fig. 6A, lower levels of the mutant proteins PipX^E4A^ and PipX^Y32A^ in comparison to PipX were detected in the later strains by western blot. In contrast, no differences in transcripts levels could be observed between mutant and control backgrounds (Fig. 6B), strongly suggesting that PipX^E4A^ and PipX^Y32A^ proteins are less stable than PipX in *S. elongatus* cells. We concluded that the effect of E4A and Y32A mutations on PipX toxicity is not attributable to up regulation of the protein levels in the mutant strains.

### PipX^E4A^ and PipX^Y32A^ can Activate Nitrogen Regulated Genes at Low 2-OG Levels

**Figure 7:**
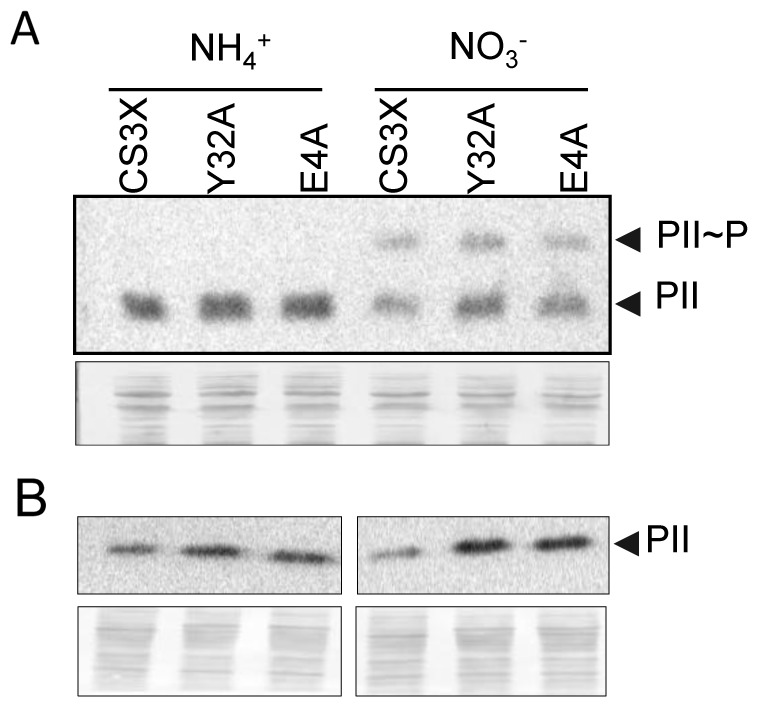
(A) Phos-tag acrylamide gel electrophoresis separation of phosphorylated and unphosphorylated P_II_. Detection of phosphorylated (P_II_∼P) and non-phosphorylated (P_II_) monomers (indicated to the right) from strains expressing wild type PipX (CS3X) and the indicated PipX variants (Y32A, E4A) grown on ammonium or nitrate as indicated. 10 µg of extract were loaded per lane on a Phos-Tag acrylamide gel and P_II_ detection was carried out using Anti-P_II_ antibody. The control of protein loading and transfer quality is shown. (B) Detection of P_II_ on protein extracts used in (A) by SDS-PAGE followed by western blot with Anti-P_II_ antibody. Other details as in A.

P_II_ is phosphorylated only when the intracellular 2-OG levels are high [32], [33], and we reasoned that the levels of P_II_∼P found in cultures where nitrogen is not limiting, particularly in ammonium-containing cultures would be an indirect but very sensitive indicator of the intracellular 2-OG status in wild type and mutant strains. We therefore examined the levels of P_II_∼P in cultures from different nitrogen regimes by Phos-tag-SDS-PAGE (see Materials and Methods) followed by western blot, a technique that, as shown in Fig. 7A, allowed a clear separation of P_II_∼P and P_II_ monomers on SDS-PAGE. Furthermore, the relative levels of phosphorylated and non-phosphorylated P_II_ in nitrate and ammonium cultures for the CS3X control strain closely agrees with those observed after native-gel separation of P_II_ trimers [34]. The slightly higher levels of the P_II_ protein detected in CS3X^E4A^ and CS3X^Y32A^ strains (Fig. 7B) are consistent with our qPCR results for the *glnB* gene (Fig. 5). Regarding CS3X^E4A^ and CS3X^Y32A^ extracts, only non-phosphorylated P_II_ was detected from ammonium cultures and the P_II_∼P/P_II_ ratio from nitrate cultures was not higher than in the control. Thus, the results showed no evidence of altered intracellular 2-OG levels in CS3X^E4A^ or CS3X^Y32A^.

## Discussion

Previous genetic analysis indicated that a small reduction in PipX levels suffices to overcome the toxic effect of PipX in *S. elongatus*. Two spontaneous point mutations (substitutions R54C and L65Q, targeting the C-terminal helices of PipX) found in different *glnB* cultures decreased activation of P*_glnN_::luxAB* and P*_glnB_::luxAB* reporters, although the molecular basis of this defect appeared different. [26]. Since we have been unable to obtain genetic evidence supporting the implication of NtcA on toxicity [25], we revisit this question using the reverse approach. We first targeted residues that are predicted to impair interactions of PipX with NtcA and/or P_II_ (E4,Y6, F12, Y32, Q34, R35, L36, Q86) as well as additional residues in interesting positions outside the interaction surfaces (D23, F38, R69, Q82), and then determined their effect on interactions with P_II_ and NtcA, toxicity and gene expression. Fig. 1 illustrates the positions of targeted residues on the PipX structure from both types of complexes (PipX-NtcA and PipX-P_II_).

**Table 1:** *S. elongatus* strains used in this work.

Strain	Genotype or relevant characteristics	Source or reference
*S. elongatus* PCC 7942	Wild-type *S. elongatus* PCC 7942	Pasteur culture collection
SA591	PipX^−^, (*pipX::*C.K1), Km^r^	[11]
CK1X^a^	PipX(Con) [Φ(C.K1-*pipX*)], Km^r^	[11]
CK1X*	PipX*(Con), Km^r^	This work
CS3X	Φ(C.S3-*pipX*), Sm^r^	[26]
CS3X*	PipX*, Sm^r^	This work
SA591-FAM2	PipX^−^, P*_glnB_::luxAB*, Km^r^ Cm^r^	[11]
CS3X-FAM2	Φ(C.S3-*pipX*), P*_glnB _::luxAB*, Sm^r^ Cm^r^	[26]
CS3X*-FAM2	PipX*, P*_glnB_::luxAB*, Sm^r^ Cm^r^	This work
SA591-FAM84W	PipX^−^, P*_glnN_::luxAB*, Km^r^ Cm^r^	[11]
CS3X-FAM84W	Φ(C.S3-*pipX*), P*_glnN_::luxAB*, Sm^r^ Cm^r^	[26]
CS3X*-FAM84W	PipX*, P*_glnN_::luxAB*, Sm^r^ Cm^r^	This work

a named as SA410 in [11].

* The asterisk represents, for CK1X strain derivatives, the following substitutions at PipX: Y6A, F12A, D23A, Q34E, R35A, L36A, F38A, R69A, Q82A and Q86A. For all CS3X derived strains it also represents substitutions E4A and Y32A.

**Table 2:** Plasmids used in this study.

Plasmid	Relevant characteristics	Source or reference
pPM128	*glnB*::C.K2, Ap^r^ Km^r^	[34]
pUAGC702	*glnB*::C.S3(+), Ap^r^ Km^r^	[25]
FAM2	P*_glnB_::luxAB* en NSII, Ap^r^ Cm^r^	[40]
FAM84W	P*_glnN_::luxAB* en NSII, Ap^r^ Cm^r^	[41]
pUAGC410	Φ(C.K1-*pipX*), Ap^r^ Km^r^	This work
pUAGC848	Φ(C.K1-*pipX* ^E4A^), Ap^r^ Km^r^	This work
pUAGC685	Φ(C.K1-*pipX* ^Y6A^), Ap^r^ Km^r^	This work
pUAGC846	Φ(C.K1-*pipX* ^F12A^), Ap^r^ Km^r^	This work
pUAGC680	Φ(C.K1-*pipX* ^D23A^), Ap^r^ Km^r^	This work
pUAGC686	Φ(C.K1-*pipX* ^Y32A^), Ap^r^ Km^r^	This work
pUAGC687	Φ(C.K1-*pipX* ^Q34E^), Ap^r^ Km^r^	This work
pUAGC688	Φ(C.K1-*pipX* ^R35A^), Ap^r^ Km^r^	This work
pUAGC847	Φ(C.K1-*pipX* ^L36A^), Ap^r^ Km^r^	This work
pUAGC849	Φ(C.K1-*pipX* ^F38A^), Ap^r^ Km^r^	This work
pUAGC689	Φ(C.K1-*pipX* ^R69A^), Ap^r^ Km^r^	This work
pUAGC850	Φ(C.K1-*pipX* ^Q82A^), Ap^r^ Km^r^	This work
pUAGC683	Φ(C.K1-*pipX* ^Q86A^), Ap^r^ Km^r^	This work
pUAGC393	Φ(C.S3-*pipX*), Ap^r^ Sm^r^	[26]
pUAGC375	Φ(C.S3-*pipX* ^E4A^), Ap^r^ Sm^r^	This work
pUAGC403	Φ(C.S3-*pipX* ^Y6A^), Ap^r^ Sm^r^	This work
pUAGC377	Φ(C.S3-*pipX* ^F12A^), Ap^r^ Sm^r^	This work
pUAGC378	Φ(C.S3-*pipX* ^D23A^), Ap^r^ Sm^r^	This work
pUAGC380	Φ(C.S3-*pipX* ^Y32A^), Ap^r^ Sm^r^	This work
pUAGC395	Φ(C.S3-*pipX* ^Q34E^), Ap^r^ Sm^r^	This work
pUAGC396	Φ(C.S3-*pipX* ^R35A^), Ap^r^ Sm^r^	This work
pUAGC397	Φ(C.S3-*pipX* ^L36A^), Ap^r^ Sm^r^	This work
pUAGC398	Φ(C.S3-*pipX* ^F38A^), Ap^r^ Sm^r^	This work
pUAGC399	Φ(C.S3-*pipX* ^R69A^), Ap^r^ Sm^r^	This work
pUAGC400	Φ(C.S3-*pipX* ^Q82A^), Ap^r^ Sm^r^	This work
pUAGC402	Φ(C.S3-*pipX* ^Q86A^), Ap^r^ Sm^r^	This work
pUAGC6	GAL4AD:NtcA, Ap^r^	[26]
pUAGC11	GAL4AD:P_II_, Ap^r^	[8]
pUAGC12	GAL4BD:P_II_, Ap^r^	[8]
pUAGC471	GAL4AD:PipX, Ap^r^	[26]
pUAGC472	GAL4BD:PipX, Ap^r^	[26]
pUAGC473	GAL4AD:PipX^E4A^, Ap^r^	This work
pUAGC474	GAL4BD:PipX^E4A^, Ap^r^	This work
pUAGC479	GAL4AD:PipX^Y6A^, Ap^r^	This work
pUAGC480	GAL4BD:PipX^Y6A^, Ap^r^	This work
pUAGC481	GAL4AD:PipX^F12A^, Ap^r^	This work
pUAGC482	GAL4BD:PipX^F12A^, Ap^r^	This work
pUAGC717	GAL4AD:PipX^D23A^, Ap^r^	This work
pUAGC718	GAL4BD:PipX^D23A^, Ap^r^	This work
pUAGC705	GAL4AD:PipX^Y32A^, Ap^r^	This work
pUAGC706	GAL4BD:PipX^Y32A^, Ap^r^	This work
pUAGC183	GAL4AD:PipX^Q34E^, Ap^r^	This work
pUAGC184	GAL4BD:PipX^Q34E^, Ap^r^	This work
pUAGC487	GAL4AD:PipX^R35A^, Ap^r^	This work
pUAGC488	GAL4BD:PipX^R35A^, Ap^r^	This work
pUAGC489	GAL4AD:PipX^L36A^, Ap^r^	This work
pUAGC490	GAL4BD:PipX^L36A^, Ap^r^	This work
pUAGC491	GAL4AD:PipX^F38A^, Ap^r^	This work
pUAGC492	GAL4BD:PipX^F38A^, Ap^r^	This work
pUAGC185	GAL4AD:PipX^R69A^, Ap^r^	This work
pUAGC186	GAL4BD:PipX^R69A^, Ap^r^	This work
pUAGC187	GAL4AD:PipX^Q82A^, Ap^r^	This work
pUAGC188	GAL4BD:PipX^Q82A^, Ap^r^	This work
pUAGC495	GAL4AD:PipX^Q86A^, Ap^r^	This work
pUAGC496	GAL4BD:PipX^Q86A^, Ap^r^	This work

Only three out of the 12 point mutations (including Q86A and controls R69A and Q82A) did not affect PipX toxicity. These three mutations did not have significant effects on gene expression (they were all in class I, Fig. 4) and only Q86A appeared slightly impaired in yeast two-hybrid signals with NtcA. The weak conservation of Q86 (located at the flexible C-terminus of PipX) in cyanobacteria suggests that it may not provide specificity for binding to NtcA, a result in agreement with the apparent wild type phenotype, for traits analyzed here, of strains carrying mutation Q86A.

A majority (7 out of 12), of the mutations examined here suppressed PipX toxicity. These mutations, resulting in PipX derivatives classified as less toxic than wild type PipX, include mutations a) abolishing all functions tested (Y6A), b) severely impairing activation of reporters and interactions signals with NtcA while moderately impairing interaction signals with P_II_ (Q34E, R35A and F38A), c) abolishing interaction signals with both P_II_ and NtcA (F12), d) abolishing interaction signals with NtcA while moderately impairing interaction signals with P_II_ (L36A) and e) only slightly impairing interaction signals with NtcA (D23A).

Since F38 is not part of the interaction surfaces with P_II_ and NtcA, the dramatic impact of mutation F38A on both, reporter activation and yeast two-hybrid signals was not anticipated from the structures of PipX-P_II_ and PipX-NtcA complexes, suggesting that F38 may play an important structural role. On the other hand, the strong impacts of Y6A and R35A mutations on reporter activation are not surprising in the light of the available structural information. Since the Y6A change did not affect expression and purification of PipX^Y6A^ for *in vitro* analyses [13] and R35A did not completely impaired interactions with P_II_ in our two hybrid assays, a major impact of these mutations on the structural integrity of PipX seems unlikely. Furthermore, yeast two-hybrid and reporter data closely agrees with the more central involvement of R35 in the interactions with NtcA than with P_II_ revealed by the PipX-NtcA and PipX- P_II_.

Our results indicate that PipX toxicity can be suppressed by a variety of point mutations at the Tudor-like domain and that some of these changes have very minor impacts on other PipX functions. In particular, the normal reporter activation of the strains carrying mutations F12A, D23A or L36A argues against up regulation by NtcA-PipX complexes as the cause of PipX toxicity. Each of these three mutations gives a distinct protein interaction pattern (Fig. 3). The integrity of F12, which is absolutely conserved in cyanobacteria, and of L36 (only L, M or I are found at this position) appeared important for interaction signals with NtcA, while that of the moderately conserved residue D23 (located in the β2–β3 hairpin, a region that appears flexible in PipX-NtcA complex) did not.

Mutations E4A and Y32A increased PipX toxicity: they did not suppress PipX toxicity in the absence of P_II_ and, importantly, they conferred lethality in a wild type background. It is worth noting here that crystal structures predicted the importance of residue Y32 for PipX-P_II_ and PipX-NtcA complexes and of E4 for PipX-P_II_ complexes, and that Surface Plasmon Resonance [13], P_II_-stimulated NAGK activity assays [13] and yeast two-hybrid analysis (Fig. S2) showed that Y32A and E4A mutations abolished interactions with P_II_. Residue Y32 is also deeply anchored into NtcA and, in agreement with this, PipX^Y32A^ is clearly defective in NtcA binding.

The finding that the toxicity conferred by PipX^Y32A^ and PipX^E4A^ proteins in *S. elongatus* was counteracted by P_II_ appears at odds with the drastic effect of E4A and Y32A mutations observed in yeast two-hybrid assays as well as *in vitro* [13]. However, the functions of PipX that are evaluated by the different assays are very different and the toxicity conferred by PipX^Y32A^ and PipX^E4A^ proteins in *S. elongatus* was counteracted, but not abolished, by P_II_. It is also important to acknowledge that PipX toxicity is gene dosage dependent, that PipX^Y32A^ and PipX^E4A^ accumulated to a lesser extent than PipX (Fig. 6), and that cellular factors in *S. elongatus* might ameliorate the impact of mutations on complex formation with P_II_. In this context, we noticed that yeast two-hybrid assays carried out under relatively permissive conditions revealed very weak, but still specific, interactions between PipX^E4A^ and P_II_ (data not shown). Thus, without underestimating the information gained by the yeast two-hybrid and *in vitro* assays, we favor the idea that both PipX^Y32A^ and PipX^E4A^ still bind, to some extent, to P_II_ *in vivo*. However, indirect genetic interactions between P_II_ and the mutant PipX proteins can not be excluded at present.

The ability of the mutant strains CS3X^E4A^ and CS3X^Y32A^ to up regulate NtcA dependent genes under nitrogen rich regimes was most intriguing. Western analyses of PipX and P_II_ proteins in these mutant strains excluded higher accumulation of PipX^E4A^ or PipX^Y32A^ *in vivo* and indicated relatively normal levels of phosphorylated P_II_, arguing against a possible unbalance of the C/N ratio. Therefore, the results indicate that both PipX^E4A^ and PipX^Y32A^ proteins stimulate gene expression when the intracellular 2-OG levels are low, raising the question of how two proteins with very different biochemical properties produce a similar outcome *in vivo*. The effect of E4A and Y32A mutations on PipX toxicity may be the combined result of altered conformation and defective binding to P_II_ in *S. elongatus*, a factor likely to increase the chances of PipX^Y32A^ and PipX^E4A^ proteins of getting involved in deleterious interactions with other cellular targets.

## Materials and Methods

### Strains and Growth Conditions

*S. elongatus* strains were routinely grown photoautotrophically at 30°C while shaking under constant illumination (40 µmol photons m^−2^ s^−1^) provided by cool white fluorescent lights. Media used were BG11_0_ (no added nitrogen), BG11 (BG11_0_ plus 17.5 mM NaNO_3_ and 10 mM HEPES/NaOH pH 7.8) and BG11_A_ (BG11_0_ plus 5 mM NH_4_Cl and 5 mM HEPES/NaOH pH 7.8). For growth on plates, the media were solidified by addition of 1% (w/v) agar. Plates were routinely incubated at 30°C under constant illumination. *S. elongatus* strains were transformed essentially as described [35]. Whenever used, antibiotic concentrations for *S. elongatus* were 10 µg kanamycin ml^−1^, 5 µg streptomycin ml^−1^ and 5 µg chloramphenicol ml^−1^. All experiments were carried out with cells grown to mid-exponential phase (OD750 nm ca. 0.5). For RNA or protein extractions aliquots of 50 ml were rapidly chilled on ice and centrifuged, and the pellets stored at −80°C.

### Yeast Two Hybrid Methods

Yeast culture and transformation procedures were performed as described [36]. For yeast two-hybrid interaction assays, derivatives of strain PJ696 carrying GAL4AD fusions were mated overnight with derivatives of strain Y187 carrying GAL4BD fusions in YPD liquid media in 96-well microtiter plates. Diploids were then analyzed for growth on different dropout media or for color development on the presence of 5-bromo-4-chloro-3-indolyl-β-D-galactopyranoside (X-Gal). The X-Gal overlay assay was performed as described previously [37]. For growth and X-Gal assays, photographs were taken after 4 days or 4–6 hours of incubation, respectively. Interaction signals between pairs of fusion proteins were determined using the three reporters present in PJ696/Y187 and classified into four different categories: +++ (very strong), ++ (relatively strong), + (weak) and – (negative).

### Construction of Plasmids and Strains

All cloning procedures were carried out in *Escherichia coli* DH5α [38] using routinely standard techniques. Constructs and mutations were analyzed by automated dideoxy DNA sequencing. Construct of strains was confirmed as described [26].

Cyanobacterial strains, plasmids and oligonucleotides used in this work are listed in Tables 1, 2 and S2. Plasmids pUAGC393, pUAGC471 and pUAGC472 were used as template for QuickChange Mutagenesis. To obtain Φ(C.K1-PipX*) derivatives, XhoI-ClaI fragments, from the indicated sources (Table S1), were cloned into pUAGC410.

### RT Reaction and Real-time PCR

Total RNA was isolated using the hot phenol method [39]. To remove genomic DNA, DNase treatment was performed on RNA samples using Turbo-DNase (Ambion Inc.) according to the manufacturer’s manual. 500 ng of total RNA were retrotranscribed in a final volume of 30 µl with the RevertAid H Minus M-MuLV reverse transcriptase (Fermentas) using a specific oligonucleotide. Real-time PCRs were perfomed using the ABI PRISM 7000 Sequence Detection System (Applied Biosystems) in 96 micro-well plates. All samples, including the non-template control, were run in duplicate. Reaction mixture contained 2 µl of cDNA obtained from retrotranscription reactions, the specific primers and the master mix Maxima™ SYBR Green qPCR. A non-template control with water was used to exclude DNA contamination. The reaction was initiated by a step at 95°C for 10 min, followed by 40 three-step amplification cycles consisting of 15 s denaturation at 95°C, 15 s annealing at 60°C and 30 s extension at 72°C. Pairs of primers were rnpB-F/rnpB-R (for *rnpB*), Sip1-BTH-F/Sip1-BTH-R (for *sipA*), GlnB-1F/GlnB-1R for (for *glnB*), GlnN-3F/GlnN-2R (for *glnN*) and nblA-F/nblA-R (for *nblA*). A final dissociation stage was run to generate a melting curve to verifiy the amplification specificity. Real-time PCR was analyzed by 7000 system SDS software (Applied Biosystems). Data were plotted as normalized reporter signals, representing the levels of fluorescence detected during the PCR process after substraction of background noise versus cycle number. A thereshold was set manually in the middle of the linear phase of the amplification curve. The cycle thereshold (*C_T_*) value is defined as the cycle in wich the increase in the reporter signal (fluorescence) cross the thereshold. The *C_T_* difference between point mutants and control strain (CS3X) samples is Δ*C_T_*. The cDNA *n*-fold change for each gene was determined by 2^−[Δ*CT*(*gene x* E4A, Y32A, Q86A)−Δ*CT*(*gene x* CS3X)]^, summarized as 2^−ΔΔCT^.

To analyse the abundance of *pipX* mRNA under nitrate growth conditions by RT-PCR, retrotranscription reactions, for *pipX* and *rnpB* with primers PipX-3X-1R and rnpB-R, respectively, were performed. A 10 µl sample of the retrotranscription reaction was subjected to 30 PCR cycles (94°C 30 s, 42°C 30 s and 72°C 30 s) with primers PipX-OV-2F and PipX-3X-1R using NETZYME DNA polymerase. For *rnpB*, 15 PCR cycles with primers rnpB-F and rnpB-R were used. For each pair of primers, a parallel reaction was carried out without reverse transcriptase to exclude DNA contamination. 10 µl of the PCR reaction were loaded on 1.5% Agarose gels to visualize the amplified products.

### Determination of Luciferase Activity

Bioluminiscence measurements were determined essentially as described [26]. Control and *pipX* point mutant strains carrying either P*_glnB_::luxAB* or P*_glnN_::luxAB* were grown until mid-exponential phase under different nitrogen regimens. Bioluminescence values obtained for the control strain CS3X (Φ(C.S3-*pipX*)) were used as reference value. Measurements were made from at least 3 independent experiments. The following bioluminescence values and standard deviation were obtained for CS3X cultures in ammonium, nitrate and 24 hours after nitrogen depletion, respectively: 2273.6±1001.9, 74300.6±4078.64 and 566390±73290.6 for P*_glnB_::luxAB* and 1274.4±388.7, 4679.3±944.6 and 100504.9±13494 for P*_glnN_::luxAB*.

### In Silico Analysis

The crystal structures of PipX bound to P_II_ or NtcA, were obtained from the protein database (PDB) with accessions 2XG8 and 2XKO, respectively. To represent structural protein data, PyMOL 0.99rc6 (DeLano Scientific LLC) was used.

### Immunodetection Methods

Immunoblot analysis of PipX was carried out as described previously [26]. Essentially cell pellets from *S. elongatus* strains were mechanically lysed with glass beads (100 nm diameter) using a minibeadbeater. After centrifugation (5000 ***g*** 3 min) the supernatant fraction was collected and protein concentrations determined by Lowry (Biorad RC DC reagent). Equal amounts of protein (60 µg) were separated in a linear gradient (5–20%) SDS-PAGE for immunoblotting. Proteins were transferred to 0.1 µm PVDF membranes (GE Healthcare) by semi-dry blot transfer, according to the manufacturer’s instructions. BSA blocked membranes were incubated o/n with Anti-PipX at a 1∶5000 dilution.

To analyze P_II_ phosphorylation, Phos-tag acrylamide gels were prepared according to the manufactureŕs instructions. Gels were loaded with 10 µg of protein extract per lane and run under constant voltage (100 V) until the dye front ran off the gel. Proteins were transferred to PVDF membrane using a semidry system. The membrane was blocked with BSA and incubated o/n in a 1∶10.000 Anti-P_II_ dilution. For antigen detection, membranes were incubated with ECL Rabbit IgG, HRP-Linked F(ab’)2 Fragment (from donkey) (GE Healthcare). Immunoreactive bands were detected using the ECL Plus Western Blotting Detection Kit (GE Healthcare) and scanning in a Typhoon 9410 fluorescence imaging system (GE Healthcare) using 488 nm/520BP40 laser/filter. To verify equal loading and transfer of proteins onto PVDF membranes, staining with Fast Green FCF dye was carried out after blotting.

## Supporting Information

Figure S1 **A stereo view of PipX in each of the two complexes is provided.**
(TIF)

Figure S2 **Yeast two hybrid interaction signals mediated by NtcA, P_II_, PipX and PipX point mutants.** Photographs show growth of diploids carrying pairs of fusion proteins on control (left panel), histidine (*HIS3*) and adenine (*ADE2*) lacking media. For each interaction assayed the GAL4 domain fused to P_II_ or NtcA is indicated. PipX point mutants are designated according to the mutated residue. Please note that the GALBD-NtcA/GALAD-PipX pair is not informative.
(TIF)

Figure S3 **Effect of** ***pipX*** **mutations on segregation of** ***glnB*** **alleles analyzed by PCR.** Detection of *glnB* alleles was carried out on several transformant clones (at least 3 independent clones after three or more consecutive transfers onto selective media) of the indicated strains carrying compatible C.K1 and C.S3 insertions. PCR products corresponding to wild type (*glnB*) and/or mutant alleles (*glnB*::C.S3 and *glnB*::C.K2) are indicated to the right (black arrowheads). Schematic representations of the different amplification products with their expected size are shown. Positions of PCR primers are indicated as black arrows. Relevant marker sizes (bp) are indicated to the left (Lane L, size marker GeneRuler 100bp plus DNA ladder, Fermentas).
(TIF)

Table S1 **Details of plasmid constructions.**
(DOCX)

Table S2 **Oligonucleotides used in this study.**
(DOCX)
