# Supplementary figures and images for: Mutational Analysis of the Cyanobacterial Nitrogen Regulator PipX
Source: PLoS One. 2012 Apr 30;7(4):e35845. doi: 10.1371/journal.pone.0035845 (PMC3340408; doi:10.1371/journal.pone.0035845)

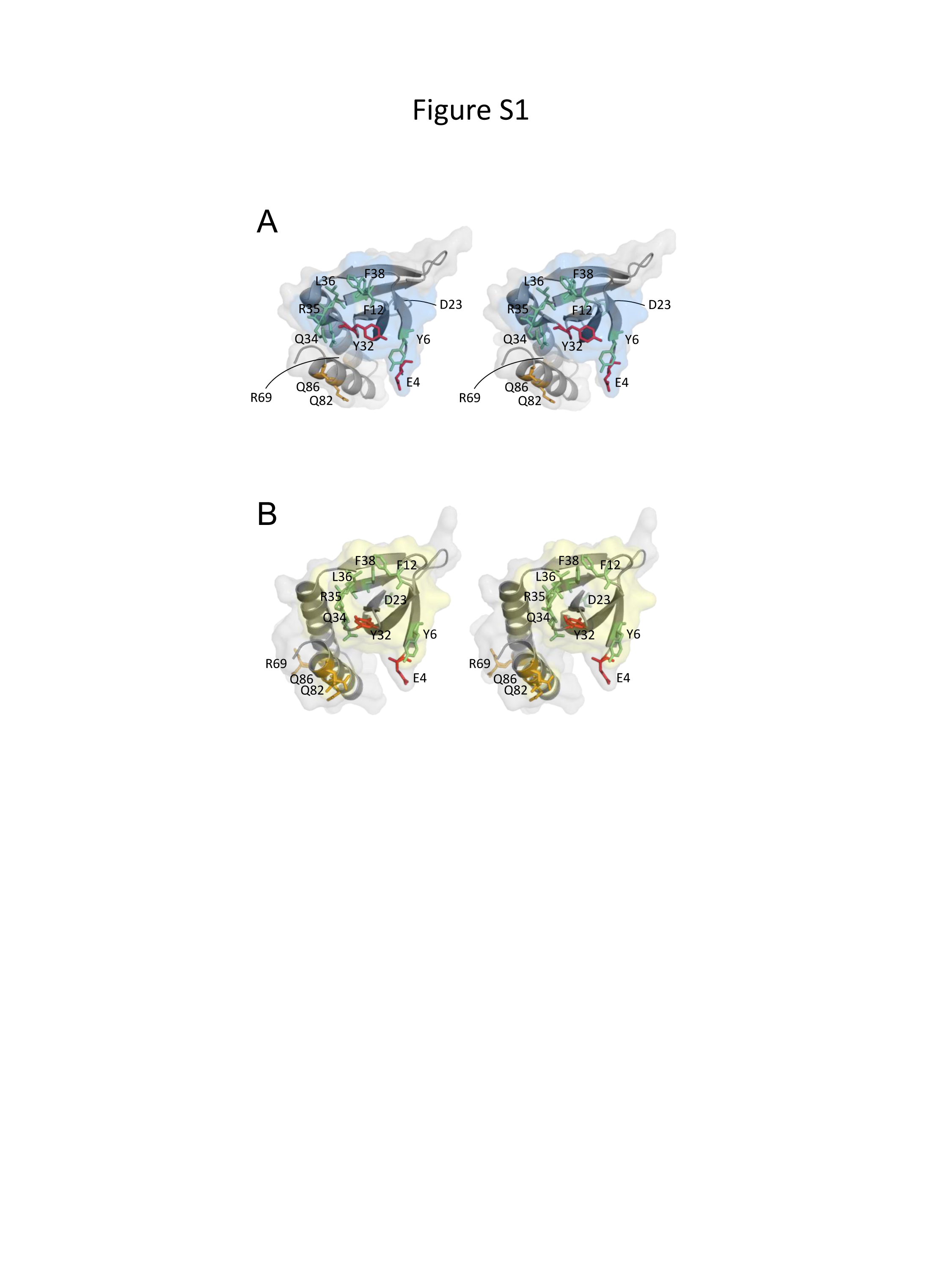

Supplement: Figure S1 — A stereo view of PipX in each of the two complexes is provided. (TIF) [file pone.0035845.s001.tif]

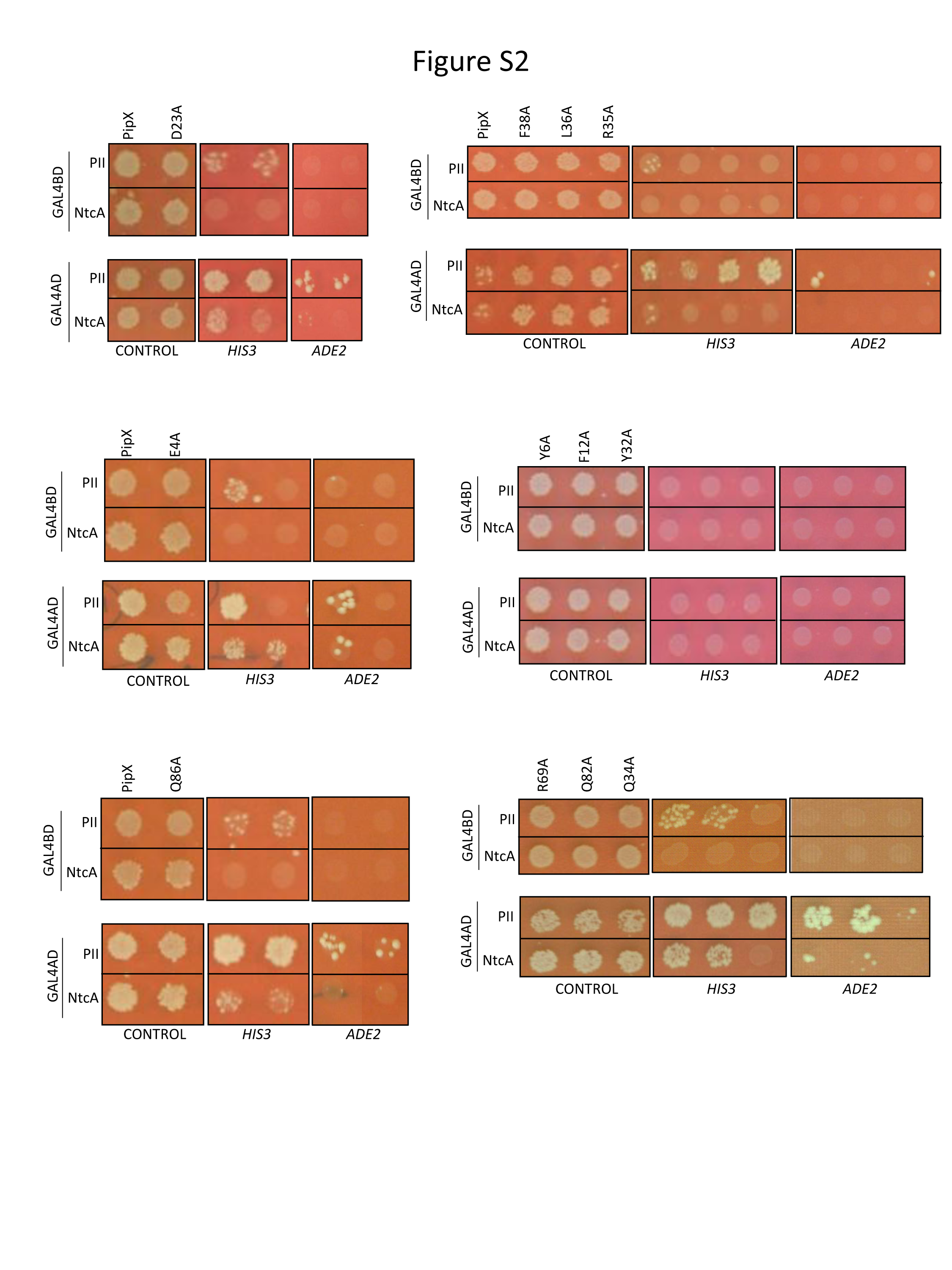

Supplement: Figure S2 — Yeast two hybrid interaction signals mediated by NtcA, PII, PipX and PipX point mutants. Photographs show growth of diploids carrying pairs of fusion proteins on control (left panel), histidine (HIS3) and adenine (ADE2) lacking media. For each interaction assayed the GAL4 domain fused to PII or NtcA is indicated. PipX point mutants are designated according to the mutated residue. Please note that the GALBD-NtcA/GALAD-PipX pair is not informative. (TIF) [file pone.0035845.s002.tif]

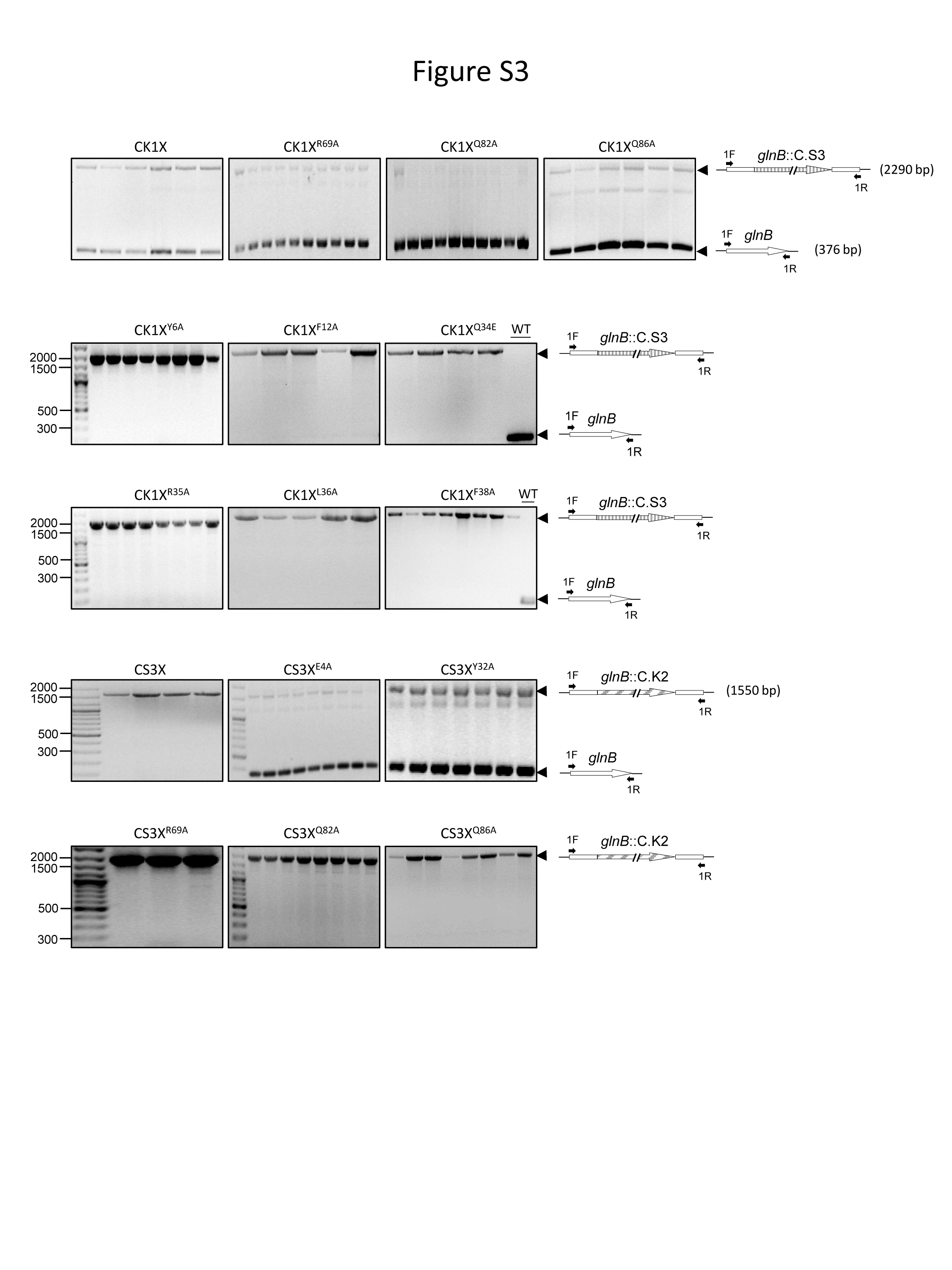

Supplement: Figure S3 — Effect of pipX mutations on segregation of glnB alleles analyzed by PCR. Detection of glnB alleles was carried out on several transformant clones (at least 3 independent clones after three or more consecutive transfers onto selective media) of the indicated strains carrying compatible C.K1 and C.S3 insertions. PCR products corresponding to wild type (glnB) and/or mutant alleles (glnB::C.S3 and glnB::C.K2) are indicated to the right (black arrowheads). Schematic representations of the different amplification products with their expected size are shown. Positions of PCR primers are indicated as black arrows. Relevant marker sizes (bp) are indicated to the left (Lane L, size marker GeneRuler 100bp plus DNA ladder, Fermentas). (TIF) [file pone.0035845.s003.tif]
