# Supplementary material for: Mutational Analysis of the Cyanobacterial Nitrogen Regulator PipX
Source: PLoS One. 2012 Apr 30;7(4):e35845. doi: 10.1371/journal.pone.0035845 (PMC3340408; doi:10.1371/journal.pone.0035845)
Supplement: Table S1 — Details of plasmid constructions. (DOCX) [file pone.0035845.s004.docx]

**TABLE S1.** Details of plasmid constructions.

| **Plasmid** | **DNA source** | **Primers or DNA fragment** |
| --- | --- | --- |
| pUAGC375 | pUAGC393 | PipX-E4A-F/PipX-E4A-R |
| pUAGC403 | pUAGC393 | PipX­Y6A­F/PipX-Y6A-R |
| pUAGC377 | pUAGC393 | PipX-F12A-F/PipX-F12A-R |
| pUAGC378 | pUAGC393 | PipX­D23A-F/PipX-D23A-R |
| pUAGC380 | pUAGC393 | PipX­Y32A­F/PipX­Y32A-R |
| pUAGC395 | pUAGC393 | PipX-Q34E-F/PipX-Q34E-R |
| pUAGC396 | pUAGC393 | PipX­R35A­F/PipX­R35A-R |
| pUAGC397 | pUAGC393 | PipX-L36A-F/PipX-L36A-R |
| pUAGC398 | pUAGC393 | PipX­F38A­F/PipX­F38A­R |
| pUAGC399 | pUAGC393 | PipX-R69A-F/PipX-R69A-R |
| pUAGC400 | pUAGC393 | PipX-Q82A-F/PipX-Q82A-R |
| pUAGC402 | pUAGC393 | PipX-Q86A-F/PipX-Q86A-R |
| pUAGC473 | pUAGC471 | PipX-E4A-F/PipX-E4A-R |
| pUAGC479 | pUAGC471 | PipX­Y6A­F/PipX-Y6A-R |
| pUAGC481 | pUAGC471 | PipX-F12A-F/PipX-F12A-R |
| pUAGC717 | pUAGC471 | PipX­D23A-F/PipX-D23A-R |
| pUAGC705 | pUAGC471 | PipX­Y32A­F/PipX­Y32A-R |
| pUAGC183 | pUAGC471 | PipX-Q34E-F/PipX-Q34E-R |
| pUAGC487 | pUAGC471 | PipX­R35A­F/PipX­R35A-R |
| pUAGC489 | pUAGC471 | PipX-L36A-F/PipX-L36A-R |
| pUAGC491 | pUAGC471 | PipX­F38A­F/PipX­F38A­R |
| pUAGC185 | pUAGC471 | PipX-R69A-F/PipX-R69A-R |
| pUAGC187 | pUAGC471 | PipX-Q82A-F/PipX-Q82A-R |
| pUAGC495 | pUAGC471 | PipX-Q86A-F/PipX-Q86A-R |
| pUAGC474 | pUAGC472 | PipX-E4A-F/PipX-E4A-R |
| pUAGC480 | pUAGC472 | PipX­Y6A­F/PipX-Y6A-R |
| pUAGC482 | pUAGC472 | PipX-F12A-F/PipX-F12A-R |
| pUAGC718 | pUAGC472 | PipX­D23A-F/PipX-D23A-R |
| pUAGC706 | pUAGC472 | PipX­Y32A­F/PipX­Y32A-R |
| pUAGC184 | pUAGC472 | PipX-Q34E-F/PipX-Q34E-R |
| pUAGC488 | pUAGC472 | PipX­R35A­F/PipX­R35A-R |
| pUAGC490 | pUAGC472 | PipX-L36A-F/PipX-L36A-R |
| pUAGC492 | pUAGC472 | PipX­F38A­F/PipX­F38A­R |
| pUAGC186 | pUAGC472 | PipX-R69A-F/PipX-R69A-R |
| pUAGC188 | pUAGC472 | PipX-Q82A-F/PipX-Q82A-R |
| pUAGC496 | pUAGC472 | PipX-Q86A-F/PipX-Q86A-R |
| pUAGC848 | pUAGC375 | XhoI-ClaI fragment |
| pUAGC685 | pUAGC403 | XhoI-ClaI fragment |
| pUAGC846 | pUAGC377 | XhoI-ClaI fragment |
| pUAGC680 | pUAGC380 | XhoI-ClaI fragment |
| pUAGC686 | pUAGC380 | XhoI-ClaI fragment |
| pUAGC687 | pUAGC395 | XhoI-ClaI fragment |
| pUAGC688 | pUAGC396 | XhoI-ClaI fragment |
| pUAGC847 | pUAGC397 | XhoI-ClaI fragment |
| pUAGC849 | pUAGC398 | XhoI-ClaI fragment |
| pUAGC689 | pUAGC399 | XhoI-ClaI fragment |
| pUAGC850 | pUAGC400 | XhoI-ClaI fragment |
| pUAGC683 | pUAGC402 | XhoI-ClaI fragment |
