# Supplementary material for: Mutational Analysis of the Cyanobacterial Nitrogen Regulator PipX
Source: PLoS One. 2012 Apr 30;7(4):e35845. doi: 10.1371/journal.pone.0035845 (PMC3340408; doi:10.1371/journal.pone.0035845)
Supplement: Table S2 — Oligonucleotides used in this study. (DOCX) [file pone.0035845.s005.docx]

**TABLE S2.** Oligonucleotides used in this study.

| **Name** | **Sequence (5’-3’)** |
| --- | --- |
| PipX­D23A-F | 5’-CCAAATCTGCAGCTTTGGGGCCAGCAAAGAACTC-3’ |
| PipX­D23A-R | 5’-GAGTTCTTTGCTGGCCCCAAAGCTGCAGATTTGG-3’ |
| PipX­E4A-F | 5´-CGAATTCGCTTCCGCGAACTACCTCAACCATCC -3’ |
| PipX­E4A-R | 5´-GGATGGTTGAGGTAGTTCGCGGAAGCGAATTCG -3’ |
| PipX-F12A-F | 5´- CCTCAACCATCCCACCGCCGGATTGCTCTACC -3’ |
| PipX-F12A-R | 5´- GGTAGAGCAATCCGGCGGTGGGATGGTTGAGG- 3’ |
| PipX­F38A-F | 5´-GCTCAGCGCCTCTTTGCTCTCGTAGCCTTTGATGC- 3’ |
| PipX­F38A-R | 5’ –GCATCAAAGGCTACGAGAGCAAAGAGGCGCTGAGC- 3’ |
| PipX-L36A-F | 5´-CCACTCTTTATGCTCAGCGCGCCTTTTTTCTCGTAGCC -3’ |
| PipX-L36A-R | 5´-GGCTACGAGAAAAAAGGCGCGCTGAGCATAAAGAGTGG- 3’ |
| PipX-Q34E-F | 5´-CTTCGCCACTCTTTATGCTGAACGCCTCTTTTTTCTCGTAG-3’ |
| PipX-Q34E-R | 5´-CTACGAGAAAAAAGAGGCGTTCAGCATAAAGAGTGGCGAAG-3’ |
| PipX-Q82A-F | 5´-CAGGAATACAACCAGCTGCAGGCGGTCTTCAAACAAACCTTTCTG-3’ |
| PipX-Q82A-R | 5´-CAGAAAGGTTTGTTTGAAGACCGCCTGCAGCTGGTTGTATTCCTG-3’ |
| PipX-Q86A-F | 5´-GCTGCAGCAAGTCTTCAAAGCAACCTTTCTGTAGCCAGC-3’ |
| PipX-Q86A-R | 5´-GCTGGCTACAGAAAGGTTGCTTTGAAGACTTGCTGCAGC-3’ |
| PipX-R35A-F | 5´- GCCACTCTTTATGCTCAGGCCCTCTTTTTTCTCGTAGCC-3’ |
| PipX-R35A-R | 5´-GGCTACGAGAAAAAAGAGGGCCTGAGCATAAAGAGTGGC-3’ |
| PipX-R69A-F | 5´-CGTCTGCGCCAGCTGGCGCGAGATGCCAGTCTG-3’ |
| PipX-R69A-R | 5´-CAGACTGGCATCTCGCGCCAGCTGGCGCAGACG-3’ |
| PipX-Y32A-1F | 5´-CGCCACTCTTGCTGCTCAGCGCC-3’ |
| PipX-Y32A-1R | 5´-GGCGCTGAGCAGCAAGAGTGGCG-3’ |
| PipX-Y6A-F | 5´-CGCTTCCGAGAACGCCCTCAACCATCCCACC-3’ |
| PipX-Y6A-R | 5´-GGTGGGATGGTTGAGGGCGTTCTCGGAAGCG-3’ |
| rnpB-F | 5´-GTGAGGAGAGTGCCACAGAA-3’ |
| rnpB-R | 5´-TAAGCCGGGTTCTGTTCTCT-3’ |
| PipX-OV-2F | 5´-GAGAATTCGCTTCCGAGAACTACC-3’ |
| PipX-3X-1R | 5´-CTGCCTCTGAATTCCTAGCTGGCTACAG-3’ |
| Sip1-BTH-F | 5´-ACCCGGATCCCTCTATGGATTTTG-3’ |
| Sip1-BTH-R | 5´-GGGGGTACCTTGATTCAGAC-3’ |
| GlnB-1F | 5´-GGCTTAAGGAGAATTCCCTTGAAGAAG-3’ |
| GlnB-1R | 5´-AACTGCAGTCGACGCTGACTTAGATTGCGTCG-3’ |
| GlnN-3F | 5´-CGAGGTGGCTCCAGG-3’ |
| GlnN-2R | 5´-GCGTTGCCAACTGACC-3’ |
| nblA-F | 5´-ATGCTCCCCCCTCTCCCCGA-3’ |
| nblA-R | 5´-CTTCCTTGGCGAATCATGCCTTTGAAG-3’ |
